# Investigating how prior knowledge influences perception and action in developmental coordination disorder

**DOI:** 10.1177/17470218231214479

**Published:** 2023-12-04

**Authors:** Kate Allen, David Harris, Tom Arthur, Greg Wood, Gavin Buckingham

**Affiliations:** 1 Department of Public Health and Sport Sciences, Faculty of Health and Life Sciences, University of Exeter, Exeter, UK; 2 Department of Health and Care Professions, Faculty of Health and Life Sciences, University of Exeter, Exeter, UK; 3 Department of Sport and Exercise Sciences, Research Centre for Musculoskeletal Science and Sports Medicine, Manchester Metropolitan University, Manchester, UK

**Keywords:** DCD, dyspraxia, grip force, size-weight illusion, object interaction

## Abstract

Developmental coordination disorder (DCD) is characterised by a broad spectrum of difficulties in performing motor tasks. It has recently been proposed that a specific deficit in sensorimotor prediction and feedforward planning might underpin these motoric impairments. The purpose of this study was to use a naturalistic object lifting paradigm to examine whether deficits in sensorimotor prediction might underpin the broad spectrum of difficulties individuals with DCD face when interacting with objects in their environment. We recruited 60 children with probable DCD and 61 children without DCD and measured perceptions of heaviness and fingertip force rate application when interacting with objects which varied in their apparent weight. If deficits in sensorimotor prediction do underpin the broad-ranging motor difficulties seen in DCD, we would expect to see a reduced effect of visual size cues on fingertip force rates and illusory misperceptions of object heaviness. We found no evidence of differences in any metrics of sensorimotor prediction between children with (*n* = 46) and without DCD (*n* = 61). Furthermore, there was no correlation between any metrics of sensorimotor prediction and motor performance (as assessed by the standard diagnostic movement assessment battery). Illusory misperceptions of object weight also did not appear to differ between groups. These findings suggest that issues with sensorimotor prediction are unlikely to affect the performance of simple real-world movements in those with DCD.

## Introduction

Developmental coordination disorder (DCD) describes a condition of poor motor performance across a broad spectrum of tasks in the absence of sensorimotor or intellectual impairment. While often co-occurring with a number of other neurodevelopmental disorders (Bolk et al., 2018), DCD is seen in approximately 5% of the otherwise typically developing population (Lingam et al., 2009). DCD typically manifests in a range of difficulties in complex visuomotor and coordinative tasks (P. H. Wilson, Ruddock, et al., 2013), from tying shoelaces to playing sports. These difficulties with planning and controlling movements usually emerge in childhood and can have a substantial impact on physical activity participation and cardio-respiratory fitness throughout adolescence (Lubans et al., 2010). These associations suggest that motor skills and hand-eye coordination can have important implications for the health of children beyond physical ability.

A major part of human behaviour is manual object interactions. Although it is easy to conceptualise our actions as being controlled entirely by our sensory systems as a movement unfolds, current models of sensorimotor control suggest that a large portion of our movements are predictive in nature (Wolpert & Flanagan, 2001). This process is thought to involve the generation of internal models to predict the outcome of our actions. Comparing these internal models to action outcomes allows (1) rapid online corrections to erroneous actions and (2) trial-by-trial learning to overcome novel environmental dynamics (Wolpert & Flanagan, 2001). Interacting with an object is a behaviour that has been shown to be particularly reliant on prediction (Flanagan & Johansson, 2011; Flanagan & Wing, 1997).

A recent model suggests that individuals with DCD might have a specific impairment in their ability to either generate these internal models and/or use them to accurately predict their action outcomes (for a recent overview, see Adams et al., 2014). This apparent impairment in predictive control has been shown in a range of sensorimotor contexts, from eye movements (Katschmarsky et al., 2001), to visually guided reaching (Wilmut et al., 2006), to selecting the most comfortable grasp orientation when gripping an object (van Swieten et al., 2010), to more complex whole-body tasks (Parr et al., 2020; Warlop et al., 2020; M. R. Wilson, Miles, et al., 2013). There have, however, been relatively few formal tests of this hypothesis in tasks which are not intrinsically difficult (i.e., free from potentially frustrating temporal constraints and/or high spatial accuracy demands).

One task that might be an effective gauge of sensorimotor prediction is that of interacting with and experiencing the weight of objects in the context of the size-weight illusion (SWI). The SWI occurs when an individual is asked to lift and judge the weight of similar-looking objects which vary in their size, but not mass (Charpentier, 1891; Nicolas et al., 2012). Participants lifting these differently sized but identically weighted objects will invariably report that the smaller objects feel heavier than the larger objects (for review, see Buckingham, 2014). By contrast, they will grip and lift the larger objects at a higher rate of force than their smaller counterparts. Both of these effects are thought to reflect different aspects of how prediction can influence our perceptual and motor systems (Brayanov & Smith, 2010). In the case of the perceptual illusion, our experience of object weight is thought to be affected by our prior expectations that the large objects will outweigh small objects and we experience a contrast to this expectation (Flanagan & Beltzner, 2000; Flanagan et al., 2008). In the context of how objects are gripped and lifted, the peak force rates, which typically occur prior to the utilisation of feedback, are also thought to be driven by prior expectations when interacting with novel objects (Gordon et al., 1991). Thus, in a typical SWI paradigm, individuals tend to grip and lift a large object with a higher rate of force, while simultaneously experiencing it as feeling less heavy, than a smaller object of the same mass. The expectations underpinning these dissociable effects appear to operate at a low level that is not necessarily consciously accessible (Buckingham, Goodale, et al., 2016; Buckingham & MacDonald, 2016), and they appear to stabilise quite early in childhood (Chouinard et al., 2019; Gordon et al., 1992).

These perceptual and motor indices of prediction are an excellent candidate to gain insight into the nuances of how a population might use, or fail to use, predictive information adequately. For example, individuals with Schizophrenia have been shown to experience a smaller SWI than controls—a finding that the authors attributed to this group’s well-established lack of feedforward processing in cognitive tasks (Williams et al., 2010). Recent work from our group has shown similar findings in terms of the prediction of grip and load forces (LFs), with the degree of feedforward prediction being inversely correlated with autistic traits in non-clinical university students (Buckingham, Michelakakis, & Rajendran, 2016). This finding is particularly relevant because autistic individuals show a range of atypicalities with integrating prior information into their perceptual judgements and motor plans in a range of contexts (Schmitz et al., 2003). Indeed, autistic children and children diagnosed with DCD show impairments in their ability to adjust their posture when they are given items to hold or have items they are holding removed (Jover et al., 2010; Mosconi et al., 2015). However, more recent work on object interaction paradigms in young autistic adults has cast doubt on the degree to which this population might differ from typically developing controls in metrics of perceptual, grip force (GF), and visuomotor coordination (Arthur et al., 2020).

The goal of our study was to examine whether children with DCD appear to have a specific deficit in the use of prior expectations in the context of the SWI. This task, which is reflective of real-world behaviour and is not inherently challenging, is particularly well-suited to examining predictive control in isolation of other motor deficits which might emerge under high indices of difficulty. This relative ease and thus lack of frustration evoked by this task have been a successful way to examine motor behaviour in a range of neurodivergent populations (Arthur et al., 2020; Buckingham et al., 2015). Given that DCD primarily manifests as a motor disorder, our primary hypothesis is that children with DCD will show a reduced tendency to initially lift large objects at a higher rate of force than equally weighted small objects (i.e., lower sensorimotor prediction). Our secondary hypothesis is that children with DCD will experience a smaller SWI than their age-matched controls. Finally, this dataset will provide exploratory insights into more general parameters of fingertip force control and haptic perception of object weight in this group.

### Materials and methods

#### Participants

A total of 121 children participated in our study; 60 of whom had probable DCD (hereafter “DCD group”) and 61 of whom did not have DCD (control group). Ethical approval for our study was granted by the University of Exeter’s Sports and Health Science Ethics Committee (171025/B/01). Participants were remunerated £20 for taking part. Recruitment took place via local primary schools in the Southwest of England (UK), social media advertisements, and word of mouth between January and November 2018. To be eligible, children had to be aged between 8 and 12 years and have no known medical conditions that affected their sensorimotor control (other than DCD). Initial inclusion criteria for the DCD group included parent/carer report of movement difficulties and a score between 15 and 55 on the revised version of the Developmental Coordination Disorder Questionnaire (DCD-Q; B. N. Wilson et al., 2009). The sample size was based on a power calculation conducted in G*Power (Faul et al., 2007) which suggested a minimum of 49 individuals per group would be required to detect an effect size of *d* = 0.74 (based on data uploaded alongside Buckingham et al., 2018), at an alpha of .05 and a power of 0.95.

On meeting these inclusion criteria, children were invited to the lab to participate in the study and their motor performance was assessed using the Movement ABC-2 Assessment Battery (MABC-2; Henderson et al., 2007). Children scoring at or below the 15th percentile were allocated to the DCD group, and children scoring above the 15th percentile were allocated to the control group, regardless of previous parent/carer reports of movement difficulties/DCD-Q. Table 1 presents an overview of the characteristics of children overall and in the DCD and control groups, respectively.

**Table 1.** Overview of participant characteristics for the overall sample, DCD group, and control group.

	Overall (*n* = 121)	DCD group (*n* = 60)	Control group (*n* = 61)
Age (mean years [*SD*])	9.5 (1.24)	9.4 (1.29)	9.6 (1.20)
Gender (% female)	41%	30%	52%
Handedness (% right-handed)	87%	85%	89%
MABC-2 (mean total score [range])	64.6 (19–103)	48.2 (19–67)	80.7 (68–103)
DCD-Q (mean [range])^*^	48.9 (17–75)	36.3 (17–69)	61.0 (26–75)
AQ (mean [range])^†^	62.1 (10–114)	73.7 (16–114)	50.6 (10–111)
ADHD Rating Scale-IV (mean [range])^†^	19.5 (0–52)	27.1 (3–52)	12.0 (0–38)

DCD: Developmental Coordination Disorder; *SD*: standard deviation; MABC-2: Movement ABC-2 Assessment Battery; DCD-Q: Developmental Coordination Disorder Questionnaire; AQ: Autism Spectrum Quotient: Children’s Version; ADHD: attention-deficit hyperactivity disorder.

* Data missing for three participants in DCD group and two participants in control group.

† Data missing for one participant in DCD group and one participant in control group.

#### Materials

Baseline/screening measures

#### Developmental Coordination Disorder Questionnaire (DCD-Q)

The DCD-Q (B. N. Wilson et al., 2009) is a well-validated, 15-item questionnaire that assesses children’s motor skills and provides an initial indication of whether a child is likely to have DCD. To complete the questionnaire, parents/carers respond to 15 motor skills statements (e.g., *“Your child throws a ball in a controlled and accurate fashion”*) using a 5-point scale from 1 *“not at all like your child”* to 5 *“extremely like your child.”* Responses are summed to create three subscale scores reflecting *control during movement*, *fine motor/handwriting*, and *general coordination*. These subscales are then summed to create a total score ranging from 15 to 75, with lower scores indicating greater movement difficulties. Scores between 15 and 55 are taken to indicate probable DCD for children aged 8–9 years of age, and scores between 15 and 57 are taken to indicate probable DCD for children aged 10–15 years of age.

#### Movement Assessment Battery for Children-2 (MABC-2)

The MABC-2 (Henderson et al., 2007) is one of the most frequently used tests in supporting a diagnosis of DCD. The MABC-2 consists of eight items divided into three sub-components: (1) manual dexterity, (2) ball skills, and (3) static and dynamic balance. Administration of the MABC-2 assessment involves presenting the child being tested with all eight items in the appropriate age band. The administration of each item involves a demonstration followed by an item-specific number of practice attempts and formal trials. Raw scores from each of the eight items are converted to standard item scores which are then summed to create a total MABC-2 test score. Total test scores ⩽ 56 are indicative of significant movement difficulties (at or below 5th percentile), scores between 57 and 67 are indicative of the child being “at risk” of having some movement difficulties (between 5th and 15th percentile) and scores > 67 are indicative of no movement difficulties (above 15th percentile).

In our study, MABC-2 scores were used to allocate children to either the DCD group (scores at or below 15th percentile) or the control group (above 15th percentile).

#### Autism Spectrum Quotient: Children’s Version (AQ)

As high levels of autistic-like traits can often co-occur with DCD, the parents of all participants completed the Autism Spectrum Quotient: Children’s Version (AQ). The AQ (Auyeung et al., 2008) is a 50-item questionnaire that assesses autistic-like traits in children aged between 4 and 11 years. Parents/carers respond to statements on their child’s behaviour (e.g., *“S/he often notices small sounds when others do not”)* on a 4-point scale from 0 *“Definitely agree”* to 3 *“Definitely disagree”*. Scoring is reverse-coded where applicable. Several different traits are assessed including social skills, attention switching, communication, and imagination. Items are summed to create a total score ranging from 0 to 150, with higher scores indicating more “autistic-like” traits.

#### ADHD Rating Scale-IV: Home Version

As certain characteristics of attention deficit hyperactivity disorder (ADHD) also co-occur with DCD, the parents of all participants completed the ADHD Rating Scale-IV: Home Version (Pappas, 2006). This scale is an 18-item questionnaire that assesses attention deficit and hyperactivity traits in children. To complete the questionnaire, parents/carers are asked to consider their child’s behaviour at home in the last 6 months and respond to 18 behaviour-related statements (e.g., *“Has difficulty sustaining attention in tasks or play activities”*) on a 4-point scale from 0 *“Never or rarely”* to 3 *“Very often”.* Specific items are summed to create subscale scores reflecting hyperactivity-impulsivity and inattention-related traits. All items are summed to create a total score ranging from 0 to 54, with higher scores indicating more attention deficit and hyperactivity traits.

#### Stimuli

To determine how prior expectations, influence perception and action, children repeatedly lifted 4 black plastic cylinders which were 7.5 cm high but varied in diameter and mass. The larger pair of objects had a diameter of 10 cm, whereas the smaller pair had a diameter of 5 cm. The heavier pair weighed 490 g, whereas the lighter pair weighed 355 g. Practice/washout trials were conducted with an intermediate-sized 490 g black plastic cylinder which was 7.5 cm tall and 7.5 cm in diameter. These stimuli have been used in previous experiments examining how prior expectations affect perception and action, as they induce a characteristic pattern of misperceptions of object weight and fingertip force errors (Arthur et al., 2020; Buckingham et al., 2018; Buckingham, Michelakakis, & Rajendran, 2016). During their first pair of lifts with the large and small objects, typically developing individuals tend to lift the large objects at a higher rate of force than the small objects (i.e., sensorimotor prediction). Perceptually, they also report the large object as feeling less heavy than the small objects (i.e., the SWI). The top surface of each object contained a t-shaped mount, which allowed for the rapid attachment and removal of a single ATI Nano17 force transducer mounted in a custom-built metal and plastic handle.

#### Procedure

Our study employed a quasi-experimental independent group design to measure performance on several metrics related to object lifting in a single lab-based testing session lasting approximately 2 hr.

All interested parents/carers, and their children, were provided with a participant information sheet, asked to complete the DCD-Q (used to provide an initial indication of whether the child was likely to have DCD), and invited to arrange a time/date to visit the lab to take part in the study.

On arrival at the lab, written informed consent and written assent were provided by parents/carers and children, respectively. Children’s movement skills were then assessed by the experimenter using the MABC-2. The MABC-2 assessment was conducted in a separate room in the presence of the parent/carer. Children and their parents/carers were then invited back into the lab for the main object-lifting experimental experiment.

The main object-lifting experiment began by asking children to sit opposite the experimenter at a large desk. The experimenter then introduced children to the equipment they would be using during the experiment (i.e., the eye-tracking glasses, motion-tracking cameras, and medium-sized object with force transducers) and described what the lifting task would involve using the following standardised script:

*“For the next bit of the study I’m going to ask you to reach out and pick up a number of objects over and over again. To do this I’d like you to sit with your hands resting on the table and focus on the sticker in the middle of the clapboard. I’ll press some buttons on the computer and then you will hear a beep. When you hear the beep, I’m going to open the clapboard and I want you to reach out and pick up the object using your thumb and first finger in a smooth, controlled, and confident fashion. Lift the object a short distance off the table and hold it steady until you hear a second beep. When you hear this second beep put the object back down and pop your hands back on the table.*

*After you’ve lifted the object, I’m going to ask you to give me a number to tell me how heavy you thought it felt. You can use any scale you like so 1 to 10 or 1 to 100 as long as big numbers mean heavier feeling objects”.*

The children were given the opportunity to adjust the height of their chair and the position of the object was adjusted accordingly to ensure that individuals could comfortably reach the object, before practicing lifting the medium-sized object five times. This allowed children to get used to the lifting procedure and prompted them to ask any questions. Following this, the experimenter set up the rest of the equipment in preparation for the main experimental trials; placing the motion sensors on the child’s dominant hand and checking these were being tracked by the motion sensor cameras. Next, a PupilLabs head-mounted eye tracker was placed on the child and a standard calibration process was performed, to examine a distinct research question related to hand–eye coordination (reported elsewhere; see Arthur et al., 2021). In the case of participants with glasses, if the child was comfortable doing so and felt able to complete the task, they removed their glasses; if they did not, they did not wear the eye tracker.

**Figure 1.**
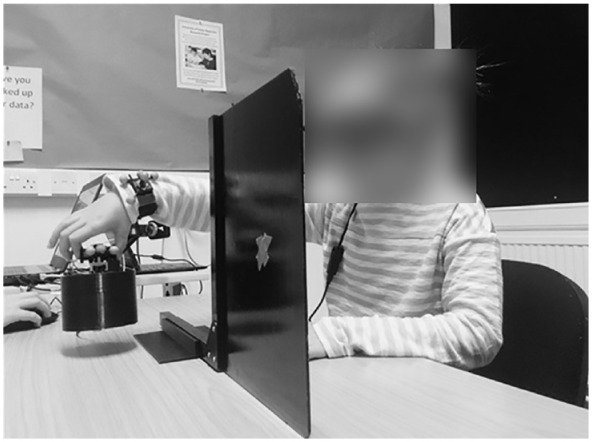
Photo of a child taking part in the experimental trials. The child is lifting a large-sized object using their thumb and forefinger.

The main experimental trials then began, starting with 5 lifts of the medium-sized 490 g object (washout trials) and then 32 lifts of the small- and large-sized heavy and light objects (experimental trials; Figure 1). The small- and large-sized objects were presented to children in 1 of 3 randomly generated orders, all of which had the same starting sequence of the aforementioned 5 washout trials with the 490 g medium-sized cylinders, then the 490 g large cylinder, and then the 490 g small cylinder. During each trial, children’s fingertip forces were measured using the ATI Nano17 force transducers and children provided unrestricted verbal heaviness ratings (Zwislocki & Goodman, 1980). Children’s hand and eye position was also recorded for a different purpose, as reported elsewhere (Arthur et al., 2021).

Parents/carers remained in the lab at all times, providing encouragement to their child where necessary but otherwise remaining out of the child’s sight. Parents/carers completed the AQ and ADHD Rating Scale-IV on a computer in the lab while their child was taking part in the main experimental trials.

#### Analysis—main

To examine sensorimotor prediction, the fingertip force data orthogonal to the surface of the grasp pad (*z*-axis) was defined as GF, and the vector sum of all remaining forces was multiplied by two and defined as LF. These force traces were then filtered with a dual-pass fourth-order Butterworth filter with a cut-off frequency of 14 Hz and differentiated with a 5-point central difference equation to yield grip force rate (GFR) and load force rate (LFR). The peak value (i.e., the largest numerical value) of each trace was identified by a custom MATLAB script and verified manually by the lead experimenter on each trial. Similar data to those examined in the current work can be found in Figure 3 of Buckingham et al. (2018). Peak grip and LFRs on the initial trials were examined in a 2 (size) × 2 (group) mixed analysis of variance (ANOVA). To directly visualise our primary hypothesis that predictive behaviour would be attenuated in children with DCD compared with the control, we calculated an index of sensorimotor prediction on the initial lifts defined as the peak GFR used on the first lift of the small heavy object subtracted from the peak GFR used on the first lift of the large heavy object (i.e., the first two experimental trials). We also examined an equivalent metric in the peak LFRs used in the first two trials. These values were then compared across the groups with separate independent-samples *t*-tests. Critical null findings were examined with Bayesian independent-samples *t*-tests, using the Cauchy prior.

To compare the magnitude of the SWI experience across the entire experiment between our two groups (our secondary hypothesis), we first transformed the experience of the illusory weight difference induced by object size into a physical magnitude, using the fact that we had objects with different mass (Hassan et al., 2020; Plaisier & Smeets, 2015). We first standardised the perception of the illusory weight difference to a rating-per-cubic centimetre value by subtracting the average rating given to the large pair of objects from the average rating given to the small pair of objects within each participant and divided this value by the difference in volume between the two stimuli (1,767.12 cm^3^). We then standardised the perception of a real weight difference to a rating-per-gram value by subtracting the average rating given to the heavy pair of objects from the average rating given to the light pair of objects within each participant, which we then divided by the weight difference between the stimuli (135 g). Finally, we divided the rating-per-cubic cm value by the rating-per-gram value to yield a single metric of each participant’s scaled SWI. This value was then compared between groups with an independent-samples *t*-test. Critical null findings were examined with Bayesian independent-samples *t*-tests, using the Cauchy prior.

Bayes factors (using a Cauchy prior) were calculated using JASP (Love et al., 2019). The Bayesian analysis was used so that conclusions were not based on a single statistical approach. Bayes factors also provide (arguably) more informative conclusions about null effects than frequentist analysis, as they indicate the relative evidence for the alternative versus null hypothesis (and vice versa). We report BF_10_ which denotes the evidence in favour of the alternative hypothesis. Values greater than one (>1) indicate the alternative to be the more likely model, while values less than one (< 1) indicate the null to be more likely. We interpret BF_10_ < 0.3 as moderate evidence in favour of the null hypothesis, and BF_10_ < 0.1 as strong evidence in favour of the null hypothesis (van Doorn et al., 2021).

#### Analysis—exploratory

To examine how fundamental grip and LF behaviour across multiple trials where objects vary in size and mass might vary between children with and without DCD, we examined average peak GF and peak LF used to lift the objects in separate 2 (size) × 2 (mass) × 2 (group) mixed ANOVAs. As these measures are less obviously related to sensorimotor prediction (Westling & Johansson, 1984), but presumably reflect some combination of outputs from the sensorimotor system, we had no predictions relating how they may differ between the groups.

## Results

### Demographics

Of the 121 children that participated in the study, 10 children with DCD failed to give appropriate perceptual reports of heaviness, yielding a sample of 111 (50 with DCD, 61 without DCD) children for the analysis of the perceptions of heaviness. For the kinetic data, 14 children with DCD failed to lift the objects in the right timeframe on one of the initial two trials, so the fingertip force analysis of sensorimotor prediction analysis was derived from 107 participants (46 with DCD, 61 without DCD).

### Main analysis

#### Sensorimotor prediction

**Figure 2.**
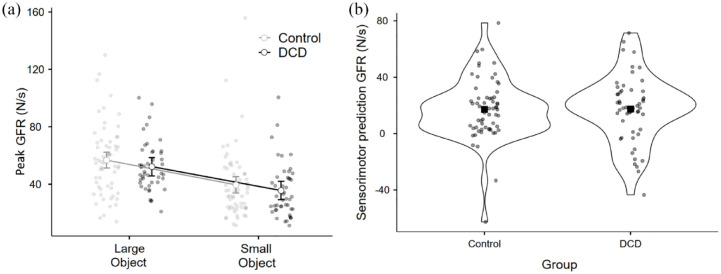
For the control (*n* = 61) and DCD (*n* = 46) groups, (a) peak grip force rates used on the initial lifts of the large and small objects. Error bars show 95% confidence intervals. (b) The indices of sensorimotor prediction directly compared between the groups for peak grip force rate with the outline of the violin plot showing density and the square showing the mean value.

In terms of GFR, there was a significant main effect of object size, *F*(1, 105) = 66.9941, *p* < .001, η^2^*p* = .39, but no main effect of group, *F*(1, 105) = 1.31, *p* = .256, η^2^*p* = .01, and no significant interaction between object size and group, *F*(1, 105) = 0.0159, *p* = .9, η^2^*p* = .0 (Figure 2a). As such, a direct comparison of sensorimotor prediction in terms of GFR between the groups yielded no significant difference, *t*(106) = 0.03, *p* = .98, *d* = 0.006 (Figure 2b). A Bayesian independent-samples *t*-test found moderate support for the null hypothesis (BF_10_ = 0.21). To provide a more stringent test of any differences between the groups, we conducted the same *t*-test comparing 34 individuals who scored in the 5th percentile or lower on the MABC-2 to the original 61 control participants (omitting participants who fell between the 5th and 10th percentiles). This test also yielded no difference between the groups, *t*(93) = 0.8, *p* = .42, *d* = 0.17, and the corresponding Bayesian independent-samples *t*-test again anecdotal/moderate support for the null hypothesis (BF_10_ = 0.299).

**Figure 3.**
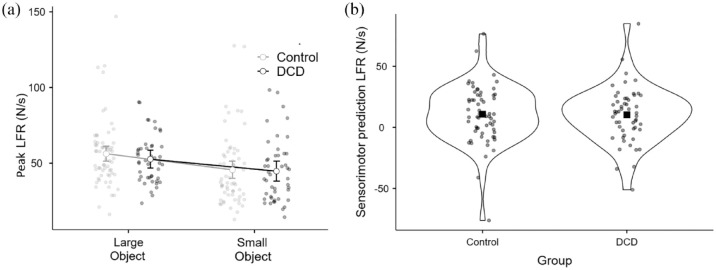
For the control (*n* = 61) and DCD (*n* = 46) groups, (a) peak load force rates used on the initial lifts of the large and small objects. Error bars show 95% confidence intervals. (b) The indices of sensorimotor prediction directly compared between the groups for peak load force rate with the outline of the violin plot showing density and the square showing the mean value.

In terms of LFR, there was a significant main effect of object size, *F*(1, 105) = 21.617, *p* < .001, η^2^*p* = .17, but no main effect of group, *F*(1, 105) = 0.389, *p* = .534, η^2^*p* = .005, and no interaction between object size and DCD, *F*(1, 105) = 0.479, *p* = .49, η^2^*p* = .004 (Figure 3a). As with GFR, a direct comparison of sensorimotor prediction in terms of LFR between the groups yielded no significant difference, *t*(106) = 0.50, *p* = .62, *d* = 0.097 (Figure 3b). A Bayesian independent-samples *t*-test found moderate support for the null hypothesis (BF_10_ = 0.22). As above, we conducted the same t-test comparing 34 individuals who scored in the 5th percentile or lower on the MABC-2 to the original 61 control participants. This test also yielded no difference between the groups, *t*(93) = 1.0 *p* = .33, *d* = 0.21, and the corresponding Bayesian independent-samples *t*-test here showed anecdotal/moderate support for the null hypothesis (BF_10_ = 0.343).

**Figure 4.**
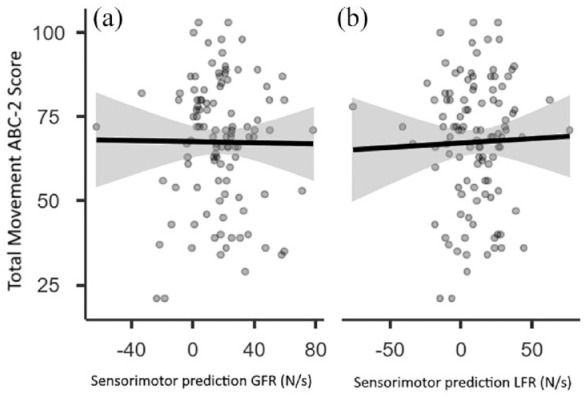
Scatterplot and regression line with confidence intervals examining how overall MABC-2 score relates to (a) peak grip force rate and (b) peak load force rate across the entire sample (*n* = 107).

Finally, to accommodate the continuous nature of the MABC-2 variable, we examined the degree to which the total MABC-2 score correlated with the indices of sensorimotor prediction for GFR and LFR across our entire sample (*n* = 107). In both cases, there was no significant correlation observed between the variables (GFR: Pearson’s *r* = –.009, *p* = .93, Figure 4a; LFR: Pearson’s *r* = .029, *p* = .77, Figure 4b). Overall, these findings provide no evidence for the proposition that children with DCD have a deficit in sensorimotor prediction compared with typically developing children.

#### Verbal heaviness ratings

**Figure 5.**
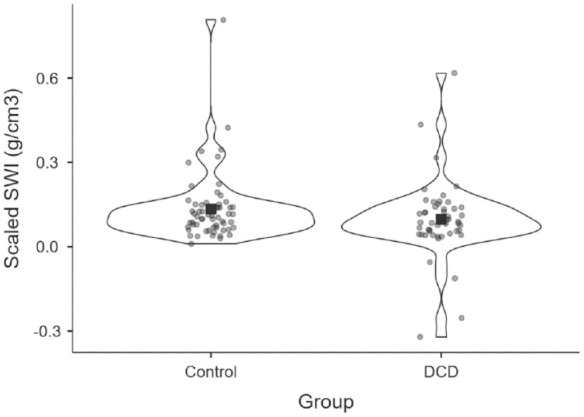
The scaled SWI compared between the control (*n* = 61) and the DCD (*n* = 50) groups. Square shows the mean value for each group, and circles show individual participants’ average magnitude of weight illusion (larger values suggest a larger SWI).

To compare scaled SWI between the groups, we conducted an independent-samples *t*-test, observing no difference between the DCD and control groups, *t*(109) = 1.56, *p* = .12, *d* = 0.297 (Figure 5). There is thus no evidence that children with DCD experience a lower SWI than typically developing children. The Bayesian independent-samples *t*-test, however, noted only anecdotal support for the null (BF_10_ = 0.596).

### Exploratory analysis

#### Grip force

**Figure 6.**
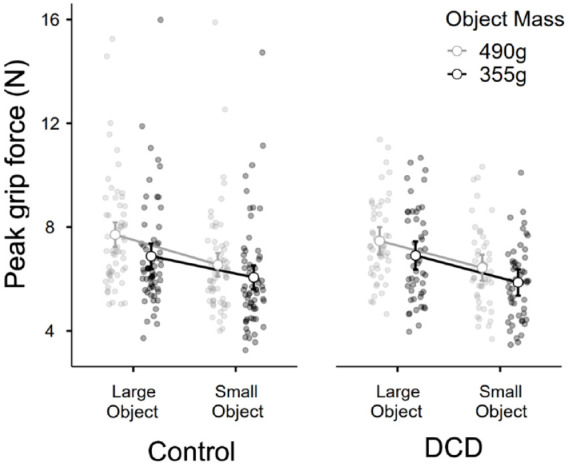
The average peak grip force used across all trials to interact with each of the objects, for the control group (*n* = 61) and the DCD group (*n* = 50).

In terms of peak GF, there was a significant main effect of object size, *F*(1, 109) = 294.9, *p* < .001, η^2^*p* = .73, a significant main effect of object mass, *F*(1, 109) = 136.5, *p* < .001, η^2^*p* = .56, but no main effect of group membership, *F*(1, 109) = 0.15, *p* = .70, η^2^*p* = .001, and no interactions between group and size, *F*(1, 109) = 0.17, *p* = .68, η^2^*p* = .002, or mass, *F*(1, 109) = 0.75, *p* = .39, η^2^*p* = .007. The three-way interaction between these variables also did not reach significance, *F*(1, 109) = 1.85, *p* = .18, η^2^*p* = .017 (Figure 6).

#### Load force

**Figure 7.**
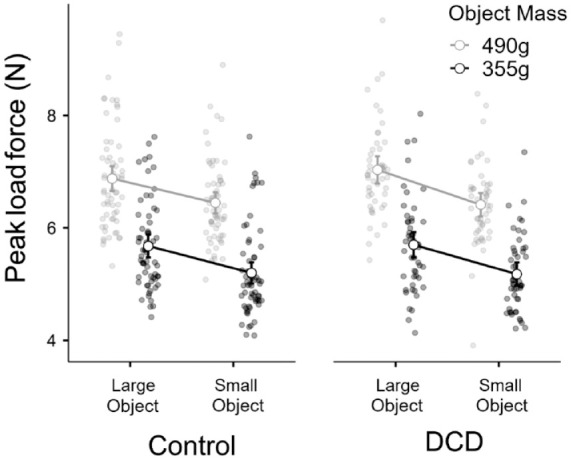
The average peak load force used across all trials to interact with each of the objects, for the control group (*n* = 61) and the DCD group (*n* = 50).

In terms of peak LF, there was a significant main effect of object size, *F* = 119.0, *p* < .001, η^2^*p* = .52, a significant main effect of object mass, *F* = 1,029.9, *p* < .001, η^2^*p* = .9, but no main effect of group membership, *F*(1, 109) = 0.05, *p* = .810, η^2^*p* < .001, and no interactions between group and size, *F* = 1.5, *p* = .22, η^2^*p* = .014], or mass, *F* = 0.61, *p* = .44, η^2^*p* = .006. The 3-way interaction between these variables also did not reach significance, *F* = 0.86, *p* = .36, η^2^*p* = .008 (Figure 7).

## Discussion

We examined whether children with DCD incorporate predictions into their sensorimotor control and perception in an atypical fashion (Adams et al., 2014). We examined this predictive behaviour in the context of an object-lifting paradigm framed around the SWI. If children with DCD have a specific impairment in using prediction to guide their actions, they would use more similar rates of force to grip and lift these objects than children without DCD. A secondary prediction was that children with DCD would also experience a smaller SWI than age-matched controls. However, in all metrics examined in this study, our data provide no evidence to support these hypotheses.

In terms of perception of the SWI—a perceptual illusion that is ostensibly driven by a contrast with prior expectations of heaviness (Baugh et al., 2012; Buckingham, 2014; Flanagan et al., 2008)—the vast majority of participants in both groups experienced this perceptual effect, and there was no statistical difference between the groups. Although 4 participants in our DCD group did show an inverted SWI (Figure 5), which could be related to their lifting dynamics (Mon-Williams & Murray, 2000), or their cognitive ability (Chouinard et al., 2019), it is more parsimonious to assume that this issue is a consequence of using a verbal metric to report perceptions of heaviness in this task, as 3 most prominently inverted SWI scores were given by children who scored above 76 on the AQ. It is worth noting, however, that we find no evidence of a reduced SWI in an adolescent population with a clinical diagnosis of autism (Arthur et al., 2020). The perceptual effect warrants further examination across larger samples of neurodivergent individuals (with multiple co-occurring conditions) to determine how motor, sensory, cognitive, and verbal factors might interact, ideally using a psychophysical which is less reliant on verbal reports such as direct comparison (Vicovaro & Burigana, 2014).

With regard to our main sensorimotor measures, we examined the tendency to grip and lift large novel objects at a higher rate of force than small novel objects, providing a clear index of sensorimotor prediction. These measures typically peak prior to object lift-off, so are thought to provide a metric of lifters’ expectations of how heavy a lifted object might be based on its visual appearance (Johansson & Flanagan, 2009). Participants in both groups showed clear evidence of sensorimotor prediction, and there was no difference between the groups—children with DCD showed the same propensity to grip and lift heavy-looking objects at a higher rate of force than light-looking objects as did children without DCD. Thus, in what we presume to be the most well-powered investigation of its kind, we find no evidence for a deficit in predictive behaviour in a simple object-lifting task in children with DCD, and moderate support to suggest that no difference exists.

The conclusion above is difficult to reconcile with the findings from Jover and colleagues’ unloading paradigm where participants must adjust their posture in a predictive fashion to maintain a stable arm posture when a load is removed from their grasp (Jover et al., 2010). However, the anticipatory postural adjustment task described by Jover et al. (2010) presumably requires significant coordination of the various body segments involved in ensuring the hand remains stable (Geuze, 2010). By contrast, the lifting while seated paradigm outlined in the current work presumably requires the coordination of fewer discrete groups of muscles than full-body tasks, so may not be as susceptible to issues surrounding coordination. The fingertip force findings presented in this article are, however, consistent with data on hand-eye coordination collected in a sub-sample of the participants during this object-lifting task, with no differences emerging between how the hand led the eye in children with and without DCD (Arthur et al., 2021). We propose that the slow-paced task of lifting an object that is mounted underneath expensive-looking equipment instrumented to facilitate a specific type of grip is the likely driver of our null findings, or simply because difficulties with DCD only emerge under duress. Studies with rapid movements and higher accuracy demands and/or more naturalistic grasping postures might highlight differences in how children with DCD interact with objects compared with typically developing children. Furthermore, is it possible that the reasonably large volume differences between stimuli may have masked any subtle differences between the groups which might have appeared with a more subtle variant of the SWI. Finally, it is worth considering that the 14 children removed from the DCD group in our analyses (due to unsuccessful completion of the task’s initial trials) may well have influenced our overall results. Indeed, their errors in performing the task may well have been associated with atypical sensorimotor prediction, and our analysis strategy could therefore be considered rather conservative in this regard.

The final point to discuss is our exploratory analysis into the peak values of grip and LF used to lift the objects on average across the entire experiment. As with the other findings in our article, we noted no clear differences between children with and without DCD. These findings contrast with those from Pereria and colleagues (2001), who noted that while children with DCD were able to adapt their fingertip forces to different surface frictions, they tended to grip objects with a higher overall force when lifting them. However, in our study, participants were presumably applying forces that were in some way related to the objects’ size and mass (which varied in a random order), whereas in the work of Pereira et al. (2001), participants were presumably optimising their fingertip forces over multiple trials in the single-mass block. Comparing random versus blocked trial orders might well be another opportunity for future work on fundamental motor properties and their interaction with sensorimotor memories and visual cues (Chouinard et al., 2005; Loh et al., 2010).

In summary, this work examined whether children with DCD performed a simple object-lifting task differently than children without DCD. In all metrics, our groups were identical, and we found no evidence for a deficit in predictive models underpinning perception or action for simple object interactions in children with DCD.
